# Achieving a High Energy Storage Performance in Grain Engineered (Ba,Sr)(Zr,Ti)O_3_ Ferroelectric Films Integrated on Si

**DOI:** 10.3390/nano15120920

**Published:** 2025-06-13

**Authors:** Fuyu Lv, Chao Liu, Hongbo Cheng, Jun Ouyang

**Affiliations:** 1 Institute of Advanced Energy Materials and Chemistry, School of Chemistry and Chemical Engineering, Qilu University of Technology (Shandong Academy of Sciences), Jinan 250353, China; lvfuyu11@163.com (F.L.); liuc@qlu.edu.cn (C.L.); hbc@qlu.edu.cn (H.C.); 2 Key Laboratory of Key Film Materials & Application for Equipments (Hunan Province), Hunan Provincial Key Laboratory of Thin Film Materials and Devices, School of Material Sciences and Engineering, Xiangtan University, Xiangtan 411105, China

**Keywords:** energy storage, dielectric capacitors, ferroelectric film, high dielectric constant, grain engineering, Si

## Abstract

BaTiO_3_-based lead-free ferroelectric films with a large recoverable energy density (*W_rec_*) and a high energy efficiency (*η*) are crucial components for next-generation dielectric capacitors, which are used in energy conditioning and storage applications in integrated circuits. In this study, grain-engineered (Ba_0.95_,Sr_0.05_)(Zr_0.2_,Ti_0.8_)O_3_ (BSZT) ferroelectric thick films (~500 nm) were prepared on Si substrates. These films were deposited at 350 °C, 100 °C lower than the temperature at which the LaNiO_3_ buffer layer was deposited on Pt/Ti. This method reduced the (001) grain population due to a weakened interface growth mode, while promoting volume growth modes that produced (110) and (111) grains with a high polarizability. As a result, these films exhibited a maximum polarization of ~88.0 μC/cm^2^, a large *W_rec_* of ~203.7 J/cm^3^, and a high energy efficiency *η* of 81.2% (@ 6.4 MV/cm). The small-field dielectric constant nearly tripled as compared with that of the same BSZT/LaNiO_3_ heterostructure deposited at the same temperature (350 °C or 450 °C). The enhanced linear dielectric response, delayed ferroelectric polarization saturation, and increased dielectric strength due to the nano-grain size, collectively contributed to the improved energy storage performance. This work provides a novel approach for fabricating high-performance dielectric capacitors for energy storage applications.

## 1. Introduction

With the rapid development of information science and technology, the market demand for ferroelectric materials has been increasing. BaTiO_3_-based ferroelectrics have some key advantages, which have attracted a lot of attention in the field of electronic materials [1]. First and foremost, it possesses excellent electrical properties, such as a high and field-tunable dielectric constant and an outstanding piezoelectric effect, which bring out a broad range of applications including capacitors, sensors, and microwave devices. In addition, they are lead-free and environmentally friendly with relatively low cost, which is in line with the development trend of modern electronic materials. Specifically, the dielectric energy storage performance has recently become a highlight of its electrical properties, showing a large recoverable energy density *W_rec_*, a high energy storage efficiency *η* and a good temperature stability in BaTiO_3_-based films [2,3,4,5,6].

The two most important evaluation criteria for the energy storage performance of a ferroelectric material are the recoverable energy density *W_rec_* and the energy efficiency *η*, both of which can be calculated from the characteristic polarization-electric field (P-E) hysteresis loop using the following relations:

(1) The recoverable energy density *W_rec_*, which is defined as $W_{rec}=\int_{P_{r}}^{P_{max}}Edp$, and the charged energy density *W_c_*, which is defined as $W_{c}=\int_{0}^{P_{max}}Edp$, where *P_r_* is the remnant polarization (polarization at zero electric field), *P_max_* is the polarization at the maximum electric field *E_max_*, and *E* is the applied electric field.
(2) The charge–discharge/energy efficiency *η*, which is defined as $\eta =\frac{W_{rec}}{W_{c}}$ [7,8].

Based on the above equations, a slim hysteresis loop is better suited to our energy storage needs [9], i.e., a P-E loop with the smallest possible remnant polarization and a delayed saturation of the electric polarization.

BaTiO_3_-based materials are widely used in capacitive energy storage applications, due to their high dielectric constants and low dielectric losses. Its microstructure can be regulated by means of doping and grain engineering to optimize its energy storage properties. Zhu et al. proposed a CMOS (complementary metal oxide semiconductor) compatible method to prepare BaTiO_3_ ferroelectric films possessing a high dielectric constant and a small remnant polarization [7,9]. In this work, to minimize grain growth and enhance the strain effect, a very thin LaNiO_3_ buffer layer (~25 nm) and BaTiO_3_ film (~160 nm) were deposited consecutively on Si. The excellent energy storage characteristics of the BaTiO_3_ film included a very large recoverable energy density of 242 J/cm^3^ and a high efficiency of 76% (at 8.75 MV/cm), which showed that suppression of grain growth is very effective for the enhancement of energy storage performance. Wang et al. used a BaTiO_3_ ceramic target doped with Zr and Sr for sputtering of (Ba,Sr)(Zr,Ti)O_3_ films. As compared to undoped BaTiO_3_ deposited at the same temperature, this material shows a lower remnant polarization, a smaller grain size, and a larger dielectric constant. A self-assembled nanostructure consisting of high-density arrays of columnar nanograins was also formed in the (Ba,Sr)(Zr,Ti)O_3_ films of ~300 nm thick, by using a buffered sputtering process at a low temperature. These columnar nanograins showed an average in-plane diameter *d* of ~20 nm and were separated by insulating non-ferroelectric grain boundaries, which acted as a periodic dead layer and further delayed the saturation of the electric polarization. These features have led to a significant increase in energy storage performance, including a high *W_rec_* of ~148 J/cm^3^ and a high energy efficiency *η* ~90%) [10]. With all these achievements, it was found that high energy storage performance in thick films (~500 nm or thicker) was rarely reported, not to mention the use of a low thermal budget for integration compatibility.

In this work, we employed an A- and B-site double-doped BaTiO_3_-based ceramic with a specific composition of (Ba_0.95_,Sr_0.05_)(Zr_0.2_,Ti_0.8_)O_3_, abbreviated as BSZT. The addition of zirconium (Zr) element plays an important role in several aspects. First, the introduction of Zr helps to optimize the microstructure of the material and reduce crystalline defects, such as dislocations and grain boundary irregularities, thus significantly improving the mechanical strength and thermal stability of the material [10,11]. Furthermore, zirconium (Zr) doping also improves the frequency stability of the dielectric constant, ensuring that the material exhibits stable dielectric properties at different frequencies while effectively reducing the dielectric loss, which is particularly important for high-frequency applications [12,13]. On the other hand, the addition of strontium (Sr) significantly improves the dielectric tunability of BaTiO_3_-based materials, i.e., the rate of change in the dielectric constant under a varying external electric field. This improvement not only enhances the potential of the material for tunable dielectric devices, but also further reduces dielectric losses and improves energy efficiency [14]. In addition, strontium (Sr) doping can also refine the grain size and form a more homogeneous microstructure, which further improves the overall performance of the material [15]. In summary, the dielectric and ferroelectric properties of BaTiO_3_-based materials have been successfully improved through the rational selection of zirconium (Zr) and strontium (Sr) as doping elements. These improvements have enabled the BSZT material to meet the requirements for a high energy storage performance.

In this work, we mainly rely on the design principles of grain engineering. Grain engineering is an advanced material design technique that optimizes properties of materials by regulating the size, shape, orientation, and distribution of grains inside the material, such as ferroelectrics [11,16,17]. Consequently, it offers a variety of possibilities for optimizing the energy storage performance of ferroelectric films. For example, by increasing the proportion of grains with a high dielectric constant, the overall energy storage property and stability of the film can be enhanced [17]. Furthermore, it was found that the use of a buffer layer is a very effective way to induce grain structures with specific crystalline orientations [18]. In order to improve the durability of thin film devices, it is imperative to deposit the films at a low temperature, thereby reducing the thermal stress and thermally induced defects (like interfacial defects due to inter-diffusion) [10,11,14,16,17]. In the present work, we chose to deposit the BSZT films on well-crystallized, (100)-oriented conductive buffer layer of LaNiO_3_ (LNO) at a low temperature (350 °C), and by doing so, we can induce a {001}-dominated, coherent interface between the two layers while maintaining a small grain size of the BSZT film. Furthermore, the deposition temperature of the LNO buffer layer was set 100 °C higher than that of the BSZT film (450 °C vs. 350 °C). The existence of this temperature difference, plus the increased film thickness (~500 nm), effectively weakens the interfacial growth mode when extending into the film bulk, i.e., it reduces the number of (001) grains from the interfacial growth mode. At the same time, it allows the bulk growth mode to dominate away from the interface, thus obtaining non-polar grains (non-polar grains refer to grains with non-polar axis orientations, whose polarizations are canted with respect to the applied field along the film normal) with a high polarizability, such as (110) and (111) grains. The addition of these grains not only enhances the dielectric constant, but also significantly elevates the dielectric strength, thus laying a solid foundation for the use of these film materials in applications such as high-performance capacitors, especially those under high-field or in a harsh environment. Overall, through this grain engineering, we have achieved a precise control of the film’s microstructure, which in turn significantly improves the dielectric properties and energy storage performance of these BSZT thick films. The latter was enhanced mainly due to a boosted dielectric constant (~340 @ 10 kHz), which led to an up-tilted P-E hysteresis loop with a large *P_max_* (~88.0 μC/cm^2^) and a high breakdown strength *E_b_* (~6.4 MV/cm). Consequently, a large recoverable energy density *W_rec_* (~203.7 J/cm^3^) and a high energy efficiency *η* (~81.2%) were obtained.

## 2. Materials and Methods

### 2.1. Materials

Single-crystalline silicon (100) substrate (10 × 10 mm) and ceramic targets of LaNiO_3_ (LNO) and (Ba_0.95_,Sr_0.05_)(Zr_0.2_,Ti_0.8_)O_3_ (BSZT) (Φ = 50 mm, L = 5 mm, 99.99%) were provided by Anhui Institute of Optics, Chinese Academy of Sciences. Platinum and titanium sputtering metal targets (Φ = 50 mm, L = 3 mm, 99.99%) was provided by Beijing (China) Goodway Metal Technology Co. The choice of Si substrate is preferred against some other commonly used semiconductor substrates (SrTiO_3_ [19,20,21,22], LaAlO_3_ [23,24], etc.) for the deposition of BaTiO_3_-based ferroelectric film capacitors, due to its dominance in current semiconductor and microelectronics industry. A very common format of Si used for the growth of perovskite ferroelectrics, like BaTiO_3_, is Pt(111)/Ti/(100)Si [25,26], which is abbreviated as Pt/Ti/Si in this study.

### 2.2. Design and Fabrication of the BSZT Films

Using a RF magnetron sputtering process, firstly, the Pt/Ti bottom electrode layer was deposited in a multi-target magnetron sputtering system with a base pressure of 2.0 × 10^−4^ Pa at a deposition temperature of 300 °C. A deposition pressure of 0.3 Pa and a pure argon atmosphere were used. The Pt/Ti/Si substrate is also commercially available. Next, a LNO buffer layer was sputtered at a deposition temperature of 450 °C, and a deposition pressure of 0.3 Pa in a mixed Ar/O_2_ atmosphere (flow rate ratio of 4:1). Lastly, a BSZT thin film was deposited on the LNO buffered Pt(111)/Ti/Si(100) substrate at a deposition temperature of 350 °C and a deposition pressure of 1.4 Pa. The deposition atmosphere was the same as that of LNO. Finally, the deposited BSZT films were cooled down in pure oxygen (2.5 Pa) at a rate of 6~8 °C/min. For electrical measurements, circular gold top electrodes (Φ = 200 μm) were deposited at room temperature using a shadow mask to form metal-ferroelectric-metal (MFM) test structures.

In our study, we employed both interfacial and bulk growth modes to optimize the microstructure of the BSZT films. The interfacial growth mode, which is dominant near the buffered substrate-film interface, leads to the formation of (001)-oriented grains aligned with the (100)-oriented LaNiO_3_ buffer layer. In contrast, the bulk growth mode, which dominates away from the interface, results in a more random distribution of grain orientations. By depositing the LaNiO_3_ buffer layer at a higher temperature (450 °C), we will promote the formation of a coherent interface with the BSZT film and the interface growth mode. By depositing the BSZT film at a lower temperature (350 °C), we will weaken the interfacial growth mode by promoting more localized, random nucleation, allowing bulk growth mode to dominate away from the interface. This will result in a large population of grains with non-polar axis orientations, such as (110) and (111) grains. These grains have higher polarizability and will contribute to an enhanced dielectric property and energy storage performance of the film.

### 2.3. Characterization Methods

The phase structure and crystalline orientation of the BSZT films were analyzed by using X-ray diffraction (XRD) in a Rigaku Dmax-2500PC diffractometer equipped with a nickel-filtered copper Kα radiation source (Rigaku, Tokyo, Japan). For estimation of the grain population by its orientation, grazing-incidence X-ray diffraction (GIXRD) was carried out in the same instrument. The nanostructure of the BSZT films was investigated via transmission electron microscopy (TEM) using a JEM-2010 microscope (JEOL, Tokyo, Japan). EDS elemental concentrations as functions of the scanning position were obtained in the same instrument by using the equipped energy dispersive X-ray spectrometer (EDS, Aztec X-Max 80T, Oxford, UK). Pseudo-static ferroelectric hysteresis loops (mono-polar and bipolar P-E loops @ 2 kHz) of the BSZT films were measured using a Multiferroic II ferroelectric tester (Radiant Technology, El Segundo, CA, USA). The electrical breakdown characteristics were analyzed via the bipolar P-E measurements and a two-parameter Weibull distribution model. Lastly, the dielectric properties of the BSZT films were measured by using a LCR meter (TH2838H, Tonghui Electronics, Chengdu, China).

## 3. Results and Discussion

Firstly, we chose to conduct our experiments in a low-temperature environment (below 500 °C), and kept the deposition temperatures of BSZT and LNO films the same on the Pt(111)/Ti/Si(100) substrate. This has been proven to be an effective approach to induce columnar nanograin arrays with a (001) texture separated by periodic grain boundaries [10,17]. The formation of this structure significantly reduces the remnant polarization of the film, thus reducing the energy loss. Furthermore, it delays the polarization saturation process and achieves a large electric polarization at high electric fields. These behaviors are essential for improving the energy storage performance. The deposition temperature *T_dep_* also has an important effect. *T_dep_* being too low leads to poor crystallinity and a small electric polarization, while too high a *T_dep_* can suppress the interface-dominated textured growth mode and promote a volume-dominated, random polycrystalline growth mode throughout the film. Such a growth will not only bring an increase in the electric polarization (both *P_r_* and *P_max_*), but also a decrease in the energy storage efficiency and a reduced dielectric breakdown strength [11,27]. Therefore, optimization of the deposition temperature is a prerequisite to take full advantage of the buffer layer effect.

**Figure 1:**
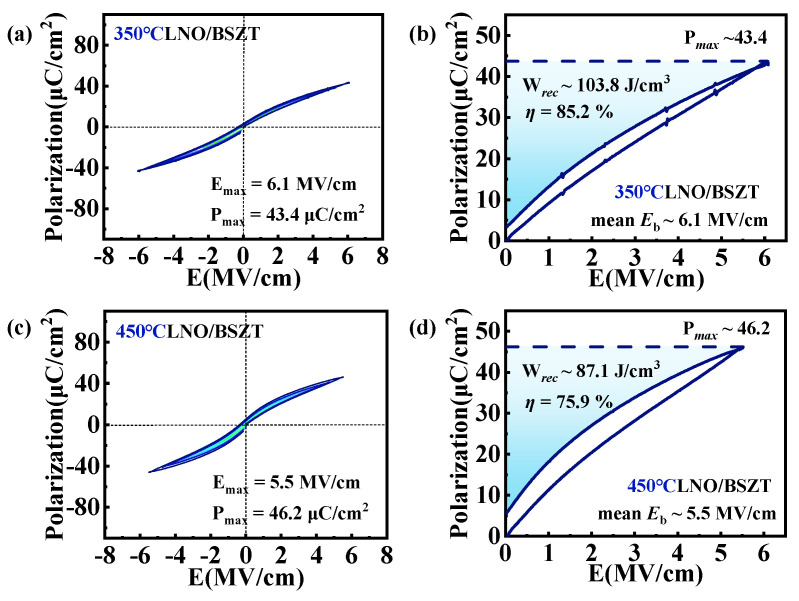
(**a**,**c**) Bipolar and (**b**,**d**) mono-polar P-E hysteresis loops of the BSZT films deposited at (**a**,**b**) 350 °C and (**c**,**d**) 450 °C, respectively. Characteristic energy storage parameters were displayed for the two films in (**b**,**d**).

In order to verify our design idea (“higher *T_dep_* for buffer layer than the ferroelectric film”, or “hot template, cold film”), we deposited LNO buffered BSZT film (~500 nm thick) on Pt/Ti coated Si substrates at 350 °C and 450 °C, respectively (the deposition temperature of both layers was the same at 350 °C or 450 °C), and measured their electrical properties. Figure 1a,c display the bipolar P-E hysteresis loops of the BSZT films deposited at 350 °C and 450 °C, respectively. From the shape of the P-E loops, it can be seen that tilted and extended hysteresis loops suitable for energy storage were obtained via these low-temperature, buffered sputtering processes. Compared with the film deposited at 450 °C, the dielectric strength *E_b_* of the film deposited at 350 °C was higher and its P-E loop were slimmer, but its maximum polarization was lower. Figure 1b,d display the mono-polar P-E loops of the two films, which simulate the charge–discharge process of the film capacitors. The energy storage parameters were obtained from these mono-polar loops, including the recoverable energy density and charge–discharge/energy efficiency *η*. It was observed that the performances of the two films were comparable with those reported in the literature [10,11,16]. The 350 °C deposited film showed a larger *W_rec_* and a higher *η* under a higher *E_b_*, while the 450 °C deposited film showed a larger polarization and a slightly higher *W_rec_* and energy storage responsivity (*W_rec_*/E) [11] at the same field as its 350 °C-deposited peer. Nevertheless, the 450 °C deposited film showed a significantly lower energy efficiency than that of the 350 °C deposited film, due to its larger remnant polarization.

**Figure 2:**
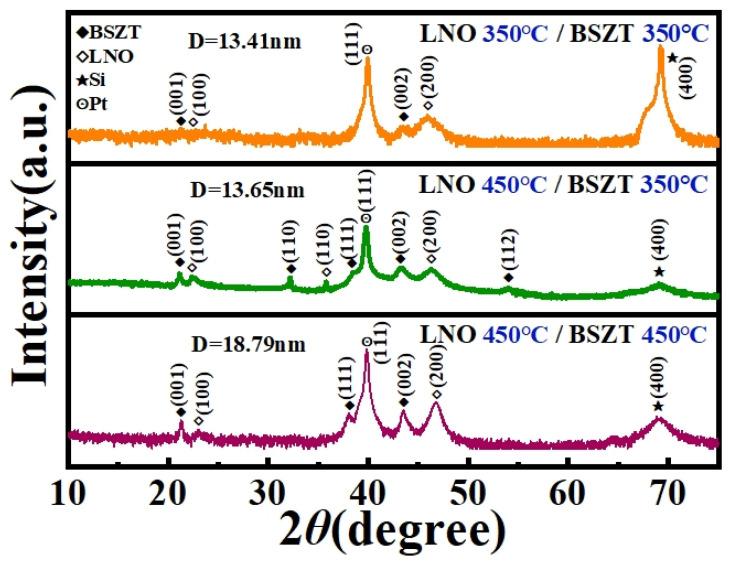
XRD 2*θ* scan patterns of the BSZT films deposited at different temperatures.

The results from Figure 1 are for buffered thin-film heterostructures dominated by interfacial growth modes, resulting in a {001} grain texture, as shown in Figure 2 (top and bottom charts). In this work, we would like to further improve the energy storage characteristics by increasing the linear dielectric contribution to *W_rec_* and *η*. The first step is to keep the buffer layer for maintaining the nanograin array structure with dominant (001)-grains near the interface, which reduces energy loss while delaying the polarization saturation. Then, the growth of grains with high dielectric constants is promoted in the film bulk. These two steps are achieved via a new strategy. The deposition temperature of the LNO buffer layer is set 100 °C higher than that of the BSZT film (“hot template, cold film”). In this way, not only the crystallinity and the growth of highly polarizable grains in the film are promoted, but the film’s small grain size and low remnant polarization are also maintained. Consequently, a larger maximum polarization (with a strong contribution from the linear dielectric part), a high energy efficiency and a high dielectric strength are simultaneously achieved. In short, the LNO/BSZT heterostructure prepared consecutively at high and low temperatures (450 °C and 350 °C) will take advantages of the energy storage characteristics of the two heterostructures uniformly deposited at one high and one low temperature, as shown in Figure 1b,d. Their respective strengths are expected to be combined to complement each other in the newly designed LNO 450 °C/BSZT 350 °C heterostructure, aiming to achieve a comprehensive optimization of the energy storage performance.

To confirm the superiority of the “hot template, cold film” design, we compared the XRD 2*θ* scan patterns of the above three heterostructures, i.e., LNO 350 °C/BSZT 350 °C, LNO 450 °C/BSZT 350 °C, and LNO 450 °C/BSZT 450 °C. Based on the positions and profiles of the (002) diffraction peaks in Figure 2, the residual strains of the three BSZT films and the size of the (002) grains were estimated (Table 1). In Figure 2, it is clearly seen that the crystallinity of the LNO 450 °C/BSZT 350 °C heterostructure is close to LNO 450 °C/BSZT 450 °C (from the intensity and sharpness of the peaks), while its calculated grain size is as small as that of LNO 350 °C/BSZT 350 °C (Table 1). The compressive residual strain of the BSZT film in the LNO 450 °C/BSZT 350 °C heterostructure is also as high as that of the BSZT film in LNO 450 °C/BSZT 450 °C (Table 1). It was reported that a compressive strain can increase the maximum polarization of a ferroelectric film [9,28], which may be attributed to an elongated out-of-plane inter-planar spacing positively coupled with the out-of-plane electric polarization. This is also beneficial for the energy storage performance of the film [9,28,29].

We estimated the residual strain and grain size of the BSZT films via XRD 2θ scan (Figure 2) and the Scherrer formula. The shift in the (002) diffraction peak position (compared to a strain-free reference), corresponds to a change in the out-of-plane lattice spacing (d) of the film, which can be obtained via Bragg’s law. Then the out-of-plane (OP) lattice strain $\varepsilon_{OP}$ can be obtained via:

$$\varepsilon_{OP}=∆d/d_{0}=(d_{film}-d_{bulk})/d_{bulk}$$

where $d_{film}$ is the measured out-of-plane lattice spacing for the film, and $d_{bulk}$ is the out-of-plane lattice spacing from the bulk reference. The in-plane strain of a film $\varepsilon_{IP}$ was estimated using $\varepsilon_{IP}=-\frac{1-\nu }{2\nu }\varepsilon_{OP}$, where ν is the Poisson’s ratio and was taken a common value of 1/3. The results (see Table 1) show compressive strain, indicating an in-plane compressive stress due to lattice mismatch between the BSZT film and the underneath Si substrate. Such a residual strain has been observed and reported in our recent publications [10,17].

Subsequently, we estimated the grain size (D) using the full width at half maximum (FWHM, β) of the XRD peak, by using the Scherrer’s formula below.

$$D=\frac{K\lambda }{\beta cos\theta }$$

where K ≈ 0.9 (Scherrer constant), and λ is the X-ray wavelength (Cu Kα, 0.154 nm) [30].

Lastly, the LNO 450 °C/BSZT 350 °C heterostructure showed a grain structure with mixed orientations, while those of the LNO 350 °C/BSZT 350 °C and LNO 450 °C/BSZT 450 °C are pre-dominantly (001) oriented. These crystalline features have met our design expectations.

**Figure 3:**
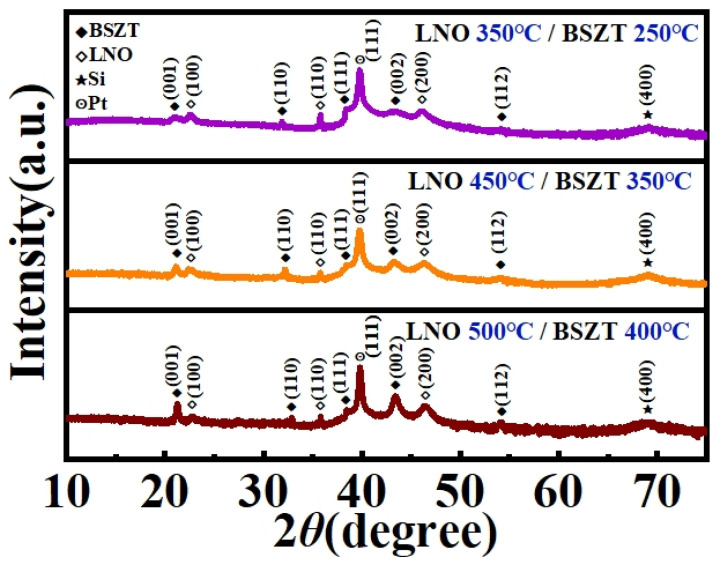
XRD 2*θ*-scan patterns of the three BSZT film samples with a “hot template, cold film” design.

In order to find the optimal deposition temperatures for our “hot template, cold film” design, three sets of variable-temperature experiments were carried out, i.e., LNO 350 °C/BSZT 250 °C, LNO 450 °C/BSZT 350 °C, and LNO 500 °C/BSZT 400 °C. The results of these three sets of experiments were compared as follows. Firstly, we performed XRD 2*θ* scans on the three film samples and the results are shown in Figure 3. Perovskite BSZT thin films of a tetragonal symmetry were identified in all three films, which showed a polycrystalline grain structure with (001)/(002), (110), (111), and (112) diffraction peaks. These observations indicate a successful design strategy of “hot template, cold film”.

**Figure 4:**
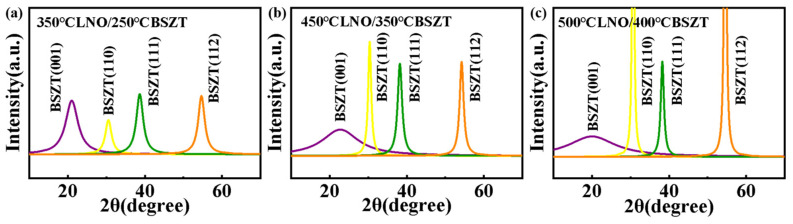
GIXD 2*θ* scans of BSZT films sputter-deposited on Pt/Ti/Si with a “hot template, cold film” design (**a**) LNO 350 °C/BSZT 250 °C, (**b**) LNO 450 °C/BSZT 350 °C, (**c**) LNO 500 °C/BSZT 400 °C.

To estimate the volume ratio of the grains with various orientations, we performed GIXD 2*θ* scans on these three film samples [31]. Figure 4 displays the fitted GIXD patterns (baseline corrected) in the 2*θ* range of [10°, 75°]. From the height of the peaks, It can be seen that, with an increasing deposition temperature, grains with non-polar orientations, whose polar axes are at an angle to the direction of the external electric field (001), i.e., “non-polar grains”, including (110), (111), and (112), showed an increasing volume ratio while grains with the (001) polar orientation displayed an decreasing volume ratio. The percentages of the grains with different orientations were estimated based on the height of their diffraction peaks in the GIXD patterns, and the results are shown in Table 2 [31]. The (001)-oriented grains decreased from ~26% in LNO 350 °C/BSZT 250 °C to ~7.6% in LNO 450 °C/BSZT 350 °C, and then to ~2.1% in LNO 500 °C/BSZT 400 °C. Such a trend indicates that the interfacial growth mode was effectively suppressed by our design of “hot template, cold film”. At the same time, this design makes the volumetric growth mode dominate in the film and promotes non-polar grains with either a high out-of-plane polarizability, such as (110) and (111) grains [7], or a boost in dielectric strength with dominated in-plane polarization component, such as the (112) grains [16]. From the grain population estimated in Table 2, it suggests that the LNO 450 °C/BSZT 350 °C heterostructure may be the optimal design point with a good combination of highly polarizable grains and a boosted dielectric strength. Overall, the grain structure of the BSZT films with the “hot template, cold film” design not only retains the high crystallinity from the high temperature deposited buffer layer and the nanograins from the low temperature deposited ferroelectric film, but is also endowed with a high dielectric constant and breakdown strength from the non-polar grain populations in (110), (111), and (112). Next, these films’ electrical properties will be measured to validate the aforementioned experimental design.

**Figure 5:**
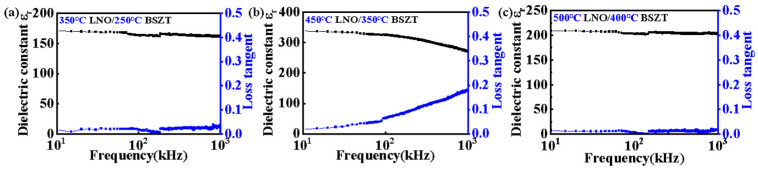
(**a**–**c**) LNO 350 °C/BSZT 250 °C, LNO 450 °C/BSZT 350 °C, LNO 500 °C/BSZT 400 °C dielectric properties (dielectric constant *ε_r_* and loss angle tangent *tgδ*) of the three BSZT thin-film heterostructures with the “hot template, cold film” design strategy, obtained in the frequency range of 10 kHz-1 MHz.

We performed frequency-dependent dielectric measurements on the three types of film samples, and the results are shown in Figure 5. The LNO 450 °C/BSZT 350 °C sample yielded the largest dielectric constant, which was about 340 at a frequency of 10 kHz, whereas the other two films yielded about 172 (LNO 350 °C/BSZT 250 °C) and 209 (LNO 500 °C/BSZT 400 °C) under the same measuring frequency. These results are consistent with the grain population estimations in Table 2, and they are superior overall against the LNO/BSZT film heterostructures deposited at the same temperature [4,7,10]. The results suggest that LNO 450 °C/BSZT 350 °C is the optimized experimental condition for obtaining the highest linear dielectric contribution for dielectric energy storage. It is noted that while the low frequency (<100 kHz) dielectric losses of the three samples are all relatively low (≤0.05), which are the key for their high energy storage performance in this frequency range, the high frequency (100 kHz to 1 MHz) dielectric losses of the LNO 450 °C/BSZT 350 °C sample are significantly higher than the other two film samples. This may be attributed to its combination of an elevated deposition temperature and a small average grain size when compared with the other two. A high deposition temperature usually leads to higher losses at high measuring frequencies (100 k to 1 MHz) due to proliferation and accelerated diffusion of charged defects, which preferentially settle at interfaces. Meanwhile, a small grain size, corresponding to a larger interface area in the film, also increases the dielectric loss in the high frequency range. Dielectric losses of the other two samples are dominated by either large grain size (500 °C LNO/400 °C BSZT) or low deposition temperature (350 °C LNO/250 °C BSZT), which have kept their values low across the whole measuring frequency range.

**Figure 6:**
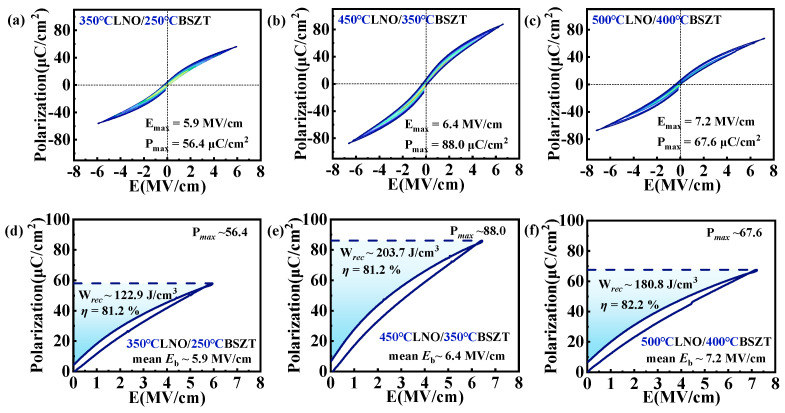
Bipolar (**a**–**c**) and mono-polar P-E hysteresis loops of (**a**,**d**) LNO 350 °C/BSZT 250 °C, (**b**,**e**) LNO 450 °C/BSZT 350 °C, and (**c**,**f**) LNO 500 °C/BSZT 400 °C, respectively. In (**d**–**f**), the corresponding recoverable energy densities, *W_rec_*, and energy efficiencies, *η*, were computed for each film. The maximum applicable electric field under which the P-E loop was measured, *E_max_* (=*E_b_*), and the maximum polarization obtained under *E_max_*, were marked out for each of the films in (**a**–**f**).

Figure 6a–c display the bi-polar P-E hysteresis loops of the three BSZT films, using a frequency of 2 kHz and the maximum electric field for each film (i.e., the breakdown electric field/dielectric strength). All three films exhibit tilted and extended P-E loops endurable to a high electric field, indicating that they all have an excellent dielectric energy storage performance. The dielectric strength *E_b_* of the films increased with the deposition temperature, from 5.9 MV/cm in LNO 350 °C/BSZT 250 °C, to 6.4 MV/cm in LNO 450 °C/BSZT 350 °C, and finally to 7.2 MV/cm in LNO 500 °C/BSZT 400 °C. This trend is consistent with the above-mentioned grain populations. Nevertheless, the LNO 450 °C/BSZT 350 °C film gives the highest maximum polarization *P_max_* ~88.0 μC/cm^2^ at a lower *E_max_*/*E_b_*, and this *P_max_* value is ~56% higher than that of the low-temperature deposited LNO 350 °C/BSZT 250 °C, and ~30% higher than that of the high-temperature deposited LNO 500 °C/BSZT 400 °C film structure. This result is consistent with the dielectric properties shown in Figure 5. Finally, based on the mono-polar P-E loops shown in Figure 6d–f, we calculated the recoverable energy density *W_rec_* and energy efficiency *η* for each film structure. Not surprisingly, LNO 450 °C/BSZT 350 °C showed the best energy storage performance with an *E_b_* ~6.4 MV/cm, a recoverable energy density *W_rec_* ~203.7 J/cm^3^ and an energy efficiency *η* reaching 81.2%. Compared with the low-temperature deposited LNO 350 °C/BSZT 250 °C, its energy storage efficiency is about the same but its *W_rec_* is about 66% (two-thirds) higher. Compared to the high-temperature deposited LNO 500 °C/BSZT 400 °C film structure, the energy efficiency of the LNO 450 °C/BSZT 350 °C film only decreases slightly (from 82.2% to 81.2%), while the energy storage density is about 13% higher at a smaller maximum electric field (6.4 MV/cm vs. 7.2 MV/cm).

**Figure 7:**
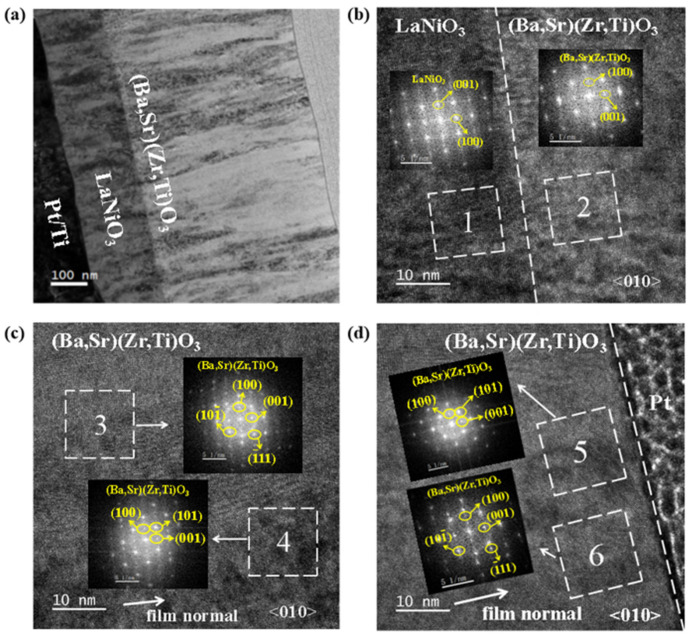
TEM analysis of the LNO450 °C/BSZT350 °C film heterostructure. (**a**) Cross-sectional low magnification TEM image, (**b**–**d**) high-resolution TEM images taken near the BSZT/LaNiO_3_ interface, in the bulk of the BSZT film, and near the film surface (Pt is the top layer pre-deposited during TEM sample preparation), respectively. In addition, the dashed boxes taken inside the LNO layer and the BSZT film in (**b**), (**c**,**d**) are the selected regions for the FFT-SAED patterns shown in the insets of (**b**–**d**), respectively. Areas 1 and 2 show a coherent, interface-dominated (100)LNO//(001)BSZT growth mode. Areas 3 and 5 show a {110}-dominated grain structure from the strong diffraction ring and spot patterns of {110}. Area 4 and 6 show a {110} and {111}-dominated, mixed grain structure, from their respective strong diffraction patterns (ring and/or spot).

We performed TEM analysis of the cross-sectional morphology and nanostructure of the LNO 450 °C/BSZT 350 °C film structure. The representative cross-sectional low- and high-resolution TEM images are shown in Figure 7a,b, respectively. In Figure 7a, the interfaces between BSZT and LaNiO_3_, and between LaNiO_3_ and Pt/Ti are smooth and clear. The thickness of the LaNiO_3_ buffer layer is about 200 nm, and the thickness of the BSZT film is about 520 nm. The BSZT film shows a microstructure consisting of columnar nanograins separated by nanometer grain boundaries, which have grown continuously from the bottom interface with the LaNiO_3_ buffer layer to the BSZT film surface. In the high-resolution TEM image shown in Figure 7b, the interface between the BSZT and LaNiO_3_ layers can be clearly seen due to elemental contrast in TEM. Figure 7b–d are high resolution TEM images taken near (b) and away from (c,d) the interfacial area between LaNiO_3_ and BSZT, respectively. The Fast Fourier Transformed Selected Area Electron Diffraction (FFT-SAED) patterns taken inside the LNO (area 1) and the BSZT layers (area 2) in (b) indicate a locally coherent growth near the interface, i.e., (100)LNO//(001)BSZT. A highly crystalline interface was revealed via the single crystal-like diffraction spots. Furthermore, as shown in Figure 7c,d in selected areas 3, 5, 4, and 6, the {110} and {111}-oriented BSZT grains were the dominant grain population in the BSZT film away from its interface with LaNiO_3_, which is consistent with the XRD results.

**Figure 8:**
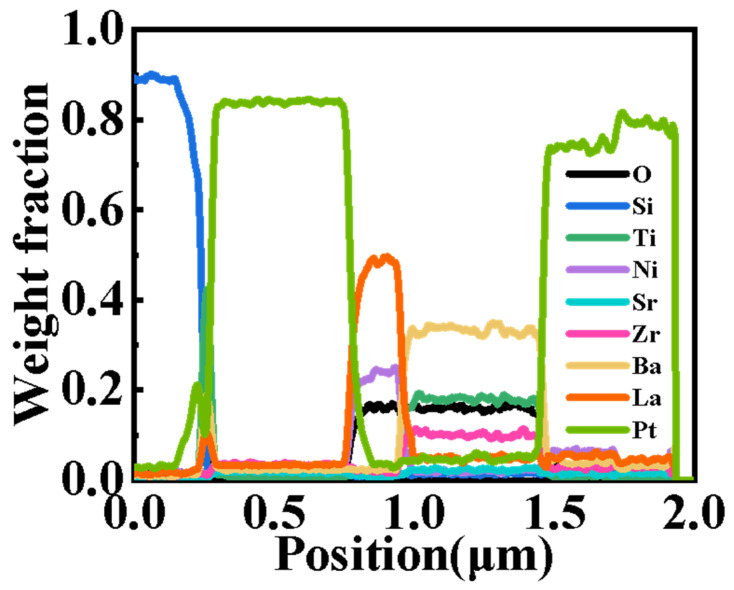
The cross-sectional energy-dispersive spectroscopy (EDS) analysis of the elemental distributions of the Si/Ti/Pt/LNO 450 °C/BSZT 350 °C/Pt film heterostructure.

A cross-sectional EDS elemental scan of the Si/Ti/Pt/LNO 450 °C/BSZT 350 °C/Pt film heterostructure was performed inside the TEM instrument, and the result is shown in Figure 8. The constituent elements, including Si, Ti, Pt, (La, Ni, O), (Ba, Sr, Zr, Ti, O), and Pt (top layer pre-deposited during TEM sample preparation), are consecutively distributed from the bottom to the surface of the film heterostructure, just as we expected. This can be attributed to a successful suppression of interlayer diffusion and decomposition of the perovskite structure by using a low-temperature sputtering process [9].

**Figure 9:**
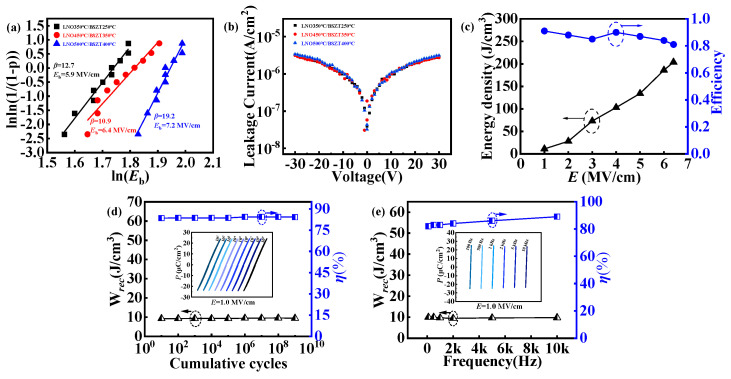
(**a**) Analysis of the breakdown electric field (*E_b_*) of the three film structures, LNO 350 °C/BSZT 250 °C, LNO 450 °C/BSZT 350 °C, and LNO 500 °C/BSZT 400 °C, using the two-parameter Weibull distribution model. (**b**) Leakage current density vs. Voltage (J-V curve) of the three films in (**a**). (**c**) The energy storage parameters (recoverable energy density *W_rec_* and efficiency *η*) as functions of the applied electric field *E*; (**d**,**e**) *W_rec_* and *η* as functions of the cumulative electric field cycle and measuring frequency (under a fixed electric field of *E* = 1.0 MV/cm) for the LNO 450 °C/BSZT 350 °C film, respectively.

To evaluate the electrical stability of the BSZT film, we carried out a Weibull distribution analysis of its dielectric strength *E_b_* and measured its leakage current density. The Weibull distribution in Figure 9a is a continuous probability distribution, which is mainly used to describe random variables in the fields of life testing, mechanical failure, reliability analysis, etc. [32]. For the dielectric strengths of the three film structures prepared under the “hot template, cold film” strategy, two-parameter Weibull distribution models were fitted to ten data points from the P-E tests under a maximum applicable electric field. The dielectric strength *E_b_* and the Weibull modulus (β) obtained from such a fitting are shown in Figure 9a [33]. The *E_b_* value of the LNO 450 °C/BSZT 350 °C film is 6.4 MV/cm, and the Weibull modulus (β) is 10.9.

The leakage current of a ferroelectric film has a multifaceted impact on their electrical performance and related applications. Specifically, it has a strong influence on the electrical polarization and P-E hysteresis loop [34,35]. Ferroelectric materials are not completely insulating as dielectrics, and when a DC voltage is applied between their top and bottom electrodes, the finite resistance of the material leads to the generation of leakage currents [35,36]. The increase in leakage current indicates the deterioration of the electrical properties of the ferroelectric films. Through Figure 9b we can see that all the three film samples, including the LNO450 °C/BSZT 350 °C film, exhibited a low leakage current density (~10^−6^ A/cm^2^ @ 30 V), which proves that the BSZT films prepared under the “hot template, cold film” strategy have a good stability for capacitive energy storage applications.

In Figure 9c, the energy storage parameters (recoverable energy density *W_rec_* and efficiency *η*) as functions of the applied electric field *E* were plotted. *W_rec_* showed a pseudo-parabola dependence on *E* while *η* decreased from ~90% at low field to ~80% in the high field end. These trends are similar to previous reports in the literature [9,10,11]. We also carried out the endurance and frequency dependent *P*-*E* measurements and extracted the associated *W_rec_* and *η* from these measurements. Figure 9d,e display *W_rec_* and *η* of the LNO 450 °C/BSZT 350 °C film as functions of the cumulative electric field cycle and measuring frequency, respectively. Under a fixed electric field of *E* = 1.0 MV/cm, the recoverable energy density (*W*_rec_) and efficiency (*η*) of the BSZT film varied very little with the number of charge–discharge cycles and the measuring frequency. The insets in Figure 9d,e show the *P*-*E* hysteresis loops measured under different cycling times and measuring frequencies (used for calculating *W_rec_* and *η*). It is noted that due to a relatively high defect concentration (as indicated by the high dielectric loss in Figure 5), the LNO 450 °C/BSZT 350 °C film failed to show similar cycling time- and frequency-independent performances at higher electric fields, nor did it yield any decent high temperature *P*-*E* hysteresis loop. This is a challenge for us to overcome in future work.

Lastly, we compared our energy density and efficiency results with other barium titanate-based thin films, as shown in Table 3. From the comparison, it can be observed that the (Ba_0.95_,Sr_0.05_)(Zr_0.2_,Ti_0.8_)O_3_ thin films, prepared using the “hot template, cold film” strategy, exhibit outstanding energy storage characteristics.

## 4. Conclusions

In this work, we optimized the energy storage performance of BSZT ferroelectric films using grain engineering. It was found that the dielectric constant, dielectric strength and recoverable energy density of the films could be significantly enhanced by engineering the size, shape, and orientation of the grains. By depositing the buffer layer and the ferroelectric film at different temperatures (100 °C higher for the former, the so called “hot template, cold film” strategy), the crystallinity, grain size, and grain orientations of the BSZT film were simultaneously optimized. The interface-dominated (00*l*)-growth mode was suppressed and the (00*l*) grains were replaced by highly polarizable (110) and (111) grains, as well as (112) grains which boost the dielectric strength *E_b_*. Consequently, a significantly enhanced energy storage performance, together with a high dielectric strength, were achieved in the LNO 450 °C/BSZT 350 °C film structure. This film exhibited a most extended P-E hysteresis loop at a high electric field (~6.4 MV/cm), achieving a giant recoverable energy density (~203.7 J/cm^3^) with a high charge–discharge efficiency (~81.2%). It also demonstrated excellent dielectric stabilities suitable for device applications. These results have laid a solid foundation for the application of barium titanate-based ferroelectric materials in high-performance energy storage capacitors.

## Figures and Tables

**Table 1:** Residual strain and grain size (via Scherrer formula) of films at different deposition temperatures (estimated with the (002) diffraction peaks).

Deposition Temperature	BSZT(002) Strain (%)	Grain Size (nm)
350 °C LNO/350 °C BSZT	−2.2	13.4
450 °C LNO/350 °C BSZT	−2.8	13.7
450 °C LNO/450 °C BSZT	−2.7	18.8

**Table 2:** Percentage of the grain population estimated via the GIXD peak heights.

BSZT Peak Percentage	(001)	(110)	(111)	(112)
LNO 350 °C/BSZT 250 °C	26%	16.5%	29.2%	28.3%
LNO 450 °C/BSZT 350 °C	7.6%	35.2%	28.6%	28.6%
LNO 500 °C/BSZT 400 °C	2.1%	42.3%	9.7%	45.9%

**Table 3:** A comparative analysis of energy storage properties between (Ba_0.95_,Sr_0.05_)(Zr_0.2_,Ti_0.8_)O_3_ thin films and other BaTiO_3_-based thin films reported in the literature.

Materials	W_rec_ (J/cm^3^)	*η* (%)	References
BaTiO_3_	135.0	80.0	[17]
Ba(Zr_0.3_Ti_0.7_)O_3_	89.9	71.6	[37]
Ba(Zr_0.35_Ti_0.65_)O_3_	65.1	72.9	[38]
Ba_0.95_La_0.05_(Zr_0.25_Ti_0.75_)O_3_	72.2	78.6	[39]
BaZr_0.35_Ti_0.65_O_3_	130.1	73.8	[40]
Ba_0.985_Na_0.015_TiO_3_	44.3	78.6	[41]
Ba(Zr_0.15_Ti_0.85_)O_3_—Ba(Zr_0.35_Ti_0.65_)O_3_	83.9	78.4	[42]
Ba_0.7_Ca_0.3_TiO_3_—BaZr_0.2_Ti_0.8_O_3_	34.8	75.1	[43]
BaTiO_3_	68.6	85.0	[44]
(Ba_0.95_,Sr_0.05_)(Zr_0.2_,Ti_0.8_)O_3_	203.7	81.2	This work

## Data Availability

Data will be made available on request.
